# Mesenchymal Stromal Cells: From Therapeutic Option to Therapeutic Target

**DOI:** 10.3390/cancers15061873

**Published:** 2023-03-21

**Authors:** Francesca Romana Stefani, Ornella Parolini, Antonietta Rosa Silini

**Affiliations:** 1 Centro di Ricerca E. Menni, Fondazione Poliambulanza Istituto Ospedaliero, 25124 Brescia, Italy; francesca.stefani@poliambulanza.it; 2 Department of Life Science and Public Health, Università Cattolica del Sacro Cuore, 00168 Rome, Italy; ornella.parolini@unicatt.it; 3 Fondazione Policlinico Universitario “Agostino Gemelli” IRCCS, 00168 Rome, Italy

As our understanding of mesenchymal stromal cells (MSC) has evolved, they have come to be recognized as an integral part of the tumor tissue, and the exploitability of their intrinsic features in the field of oncology has reached a standstill. Currently, there are 1621 registered clinical trials (clinicaltrials.gov) on “mesenchymal cells”, and yet none of them are exploring or explored unmodified MSC as a therapeutic option for cancer. Indeed, the therapeutic potential of these cells in oncology relies on their ability to migrate towards sites of injury and inflammation and function as delivery systems for the local release of therapeutics [1], recently reported as also occurring via exosomes [2].

Since the first characterization of these cells in healthy bone marrow (BM) by Friedenstein, MSC have been described in various tissues of healthy and diseased bodies, performing a plethora of functions mostly aimed towards the governance of tissue homeostasis.

When considering pathological conditions and cancer, in particular, evidence suggests that the ability of local or recruited MSC to maintain a steady state is compromised, and the cells become integrated into the newly formed organ at the expense of its healthy counterpart. Cancer-associated MSC (CA-MSC) have been shown to promote multi-organ metastasis and to govern tumor immune surveillance [3]. Furthermore, BM-derived cancer-associated fibroblasts (CAFs) promote angiogenesis in breast cancer [4] and exhibit a unique inflammatory profile depending on the location. Indeed, several studies have highlighted that the microenvironment can reprogram stromal cells and inflammatory cytokines released in the tumor microenvironment (TME), ultimately boosting the immune-suppressive properties of MSC and favoring tumor growth [5,6].

This Special Issue, entitled “Role of Mesenchymal Stromal Cells (MSC) in Cancer Progression and Cancer Therapy”, aims to further explore the fate of MSC within malignancies, encompassing the crosstalk between the stroma and the tumor as well as that between the different stromal components.

A crucial phase of cancer progression is the ability of the tumor to communicate with its surroundings. To this end, Aasebø and colleagues dissected the bidirectional communication between MSC and acute myeloid leukemia (AML) cells [7]. The authors performed a proteomic analysis of AML cells isolated from 40 patients and evaluated how healthy MSC influence the proteomic pattern of cancer cells. The results showed that, overall, patient heterogeneity was maintained upon challenge with MSC; nevertheless, the authors observed a reduction in patient heterogeneity for a minority of proteins, including extracellular matrix molecules, proteases (i.e., protein modifiers) and soluble adhesion molecules, thus highlighting possible targetable pathways. In line with this study, Fallati and colleagues reviewed the ability of MSC to orchestrate a leukemia-supportive microenvironment [8]. The authors detailed the capacity of the cells to contribute to the different stages of cancer and provided an overview of the potential mechanisms of action. They described the possible contribution of MSC to leukemogenesis and the cells’ ability to generate a leukemia-permissive environment by acting directly on tumor cells and indirectly via the microenvironment. Lastly, the authors reviewed the mechanisms of chemoprotection that involve MSC and are based upon a bi-directional exchange of soluble factors (metabolites, amino acids, etc.), extracellular vesicles (EVs) and nanotube-based connections. The emergence of chemoresistance is, indeed, a major clinical problem for tumors in cases where chemotherapy remains the frontline treatment. Železnik Ramuta and colleagues reviewed 42 studies published between 2001 and 2022 evaluating the role of MSC in chemoresistance [9]. The results highlighted the existence of various mechanisms involved in this specific function of MSC, most of them affecting signaling pathways related to apoptosis and proliferation. Sentek and colleagues illustrated the relevance of the niche to interactions between endogenous lung-resident mesenchymal stem cells (LRMSC) and tumor cells [10]. They described peculiar features of “cancer-educated” LR-MSCs and discussed their potential to differentiate into CAFs and pericytes and, ultimately, favor tumor progression. Papait and colleagues acknowledged the role of CAFs in cancer development, addressing the main open questions regarding these controversial cells [11]. They clarified different aspects related to phenotype identification and subtype specification and discussed some of the most advanced technologies involved in the process. Finally, they reviewed pre-clinical and clinical attempts to target CAFs in various types of cancer. As cancer progression is also defined by stroma-to-stroma interactions, Çakır and colleagues investigated the capacity of melanoma-associated fibroblasts (MAFs) to modulate macrophage functions [12]. The authors observed that the MAFs were able to shape the functional phenotype of macrophages and elicit IL-10 secretory production in these cells via both the cyclo-oxygenase pathway and IDO, thus regulating tumor immunity.

With their innate features having been unraveled, more questions will be raised concerning the safety and translational relevance of MSC in cancer. This Special Issue highlights the need for a deeper understanding of the fundamental processes that regulate MSC biology in health and disease to develop a clinically relevant therapeutic strategy that takes into consideration the recipient’s environment as a bi-directional type of communication.
